# Duplication Cyst of the Sigmoid Colon

**DOI:** 10.1155/2009/918401

**Published:** 2010-02-09

**Authors:** Bastian Domajnko, Rabih M. Salloum

**Affiliations:** Department of Surgery, University of Rochester Medical Center, 601 Elmwood Avenue, Rochester, NY 14642, USA

## Abstract

A 21-year-old male with developmental delay presented with abdominal pain of two days' duration. He was afebrile and his abdomen was soft with mild diffuse tenderness. There were no peritoneal signs. Plain x-ray demonstrated a large air-filled structure in the right upper quadrant. Computed tomography of the abdomen revealed a 9 × 8 cm structure adjacent to the hepatic flexure containing an air-fluid level. It did not contain oral contrast and had no apparent communication with the colon. At operation, the cystic lesion was identified as a duplication cyst of the sigmoid colon that was adherent to the right upper quadrant. The cyst was excised with a segment of the sigmoid colon and a stapled colo-colostomy was performed. Recovery was uneventful. Final pathology was consistent with a duplication cyst of the sigmoid colon. The cyst was attached to the colon but did not communicate with the lumen.

## 1. Case Report

**Figure 1:**
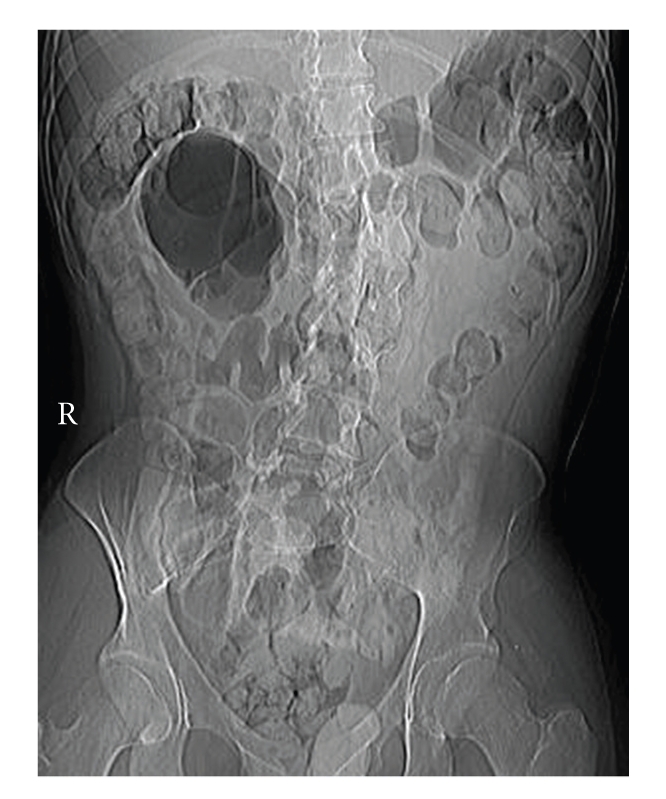
Abdominal X-ray demonstrating a cystic air-filled structure in the right upper quadrant.

**Figure 2:**
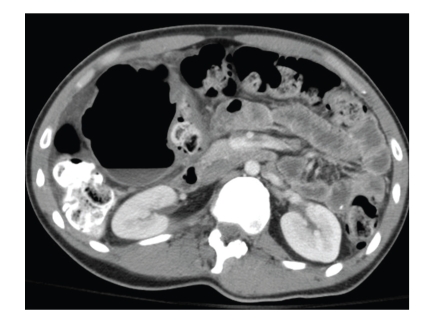
Computed tomography of the abdomen demonstrating the cyst containing an air-fluid level.

A 21-year-old male with developmental delay presented with abdominal pain of two days' duration. He was afebrile and his abdomen was soft with mild diffuse tenderness. There were no peritoneal signs. Plain X-ray demonstrated a large air-filled structure in the right upper quadrant (Figure 1). Computed tomography (CT) of the abdomen revealed a 9 × 8 cm structure adjacent to the hepatic flexure containing an air-fluid level (Figure 2). It did not contain oral contrast and had no apparent communication with the colon.

**Figure 3:**
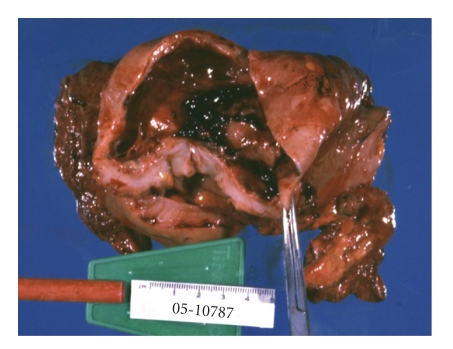
The duplication cyst opened, containing a small amount of blood clots.

**Figure 4:**
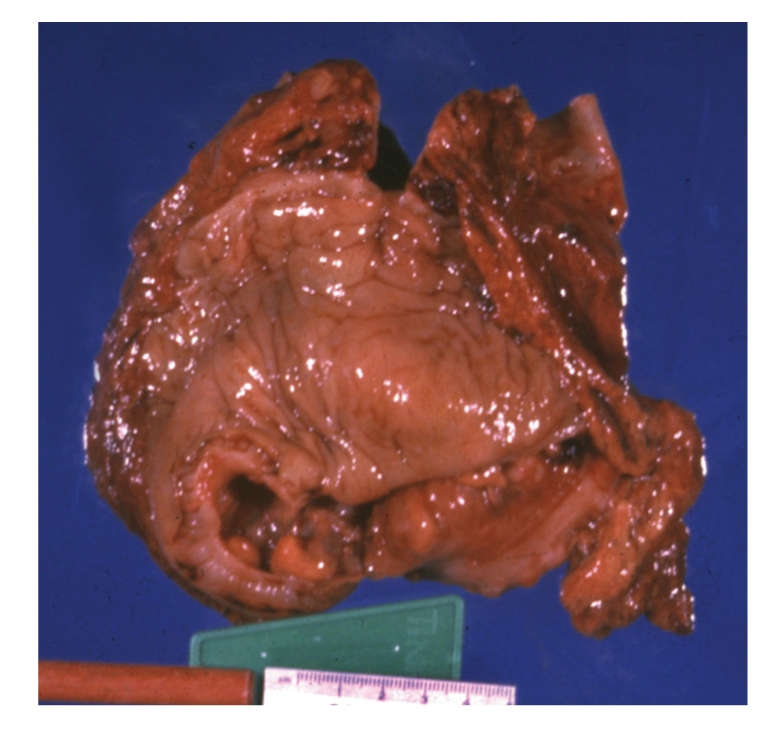
The lumen of the sigmoid colon sharing a common wall with the duplication cyst but without direct communication.

At operation, the cystic lesion was identified as a duplication cyst of the sigmoid colon that was adherent to the right upper quadrant. The cyst was excised with a segment of the sigmoid colon and a stapled colocolostomy was performed. Recovery was uneventful. Final pathology was consistent with a duplication cyst of the sigmoid colon (Figure 3). The cyst was attached to the colon (Figure 4) but did not communicate with the lumen.

## 2. Discussion

Duplications of the gastrointestinal tract are rare congenital anomalies that can occur anywhere throughout the gastrointestinal tract. They share a common wall with an adjacent portion of the gastrointestinal tract, but may or may not have a communication with the bowel. The cyst has at least one outer muscular layer and is lined with varying types of gastrointestinal mucosa; gastric mucosa may be found in over 50% of duplications including the colon and rectum [1]. Duplications are also characterized as cystic (86%) or tubular in appearance. The most common site for duplications is the ileum, accounting for over 60% of cases [1]. Duplications may also be found in the esophagus, duodenum, and stomach. Duplication of the colon is a rare abnormality, comprising only 6.8% of all gastrointestinal duplications in a recent series of 73 patients [1]. Fewer than 100 cases of colonic duplication have been reported in the literature [2]. Complete colorectal duplication has also been described [3].

The clinical presentation of gastrointestinal duplications varies with the age of the patient. The majority of cases present before the age of 2 years, and with the use of prenatal ultrasound these lesions are becoming more frequently identified in utero. Infants and neonates usually present with vomiting, abdominal distention, pain, or the presence of an abdominal mass. Most colonic duplication cysts remain asymptomatic however, and may not be diagnosed until adulthood. When symptomatic, abdominal pain is the most common presentation [4]. Other clinical manifestations of colonic duplications include obstruction, volvulus, and bleeding [2]. Gastrointestinal hemorrhage has been reported in cases in which ectopic gastric mucosa ulcerates and erodes into adjacent organs or vessels. Malignant degeneration of the duplication cyst is a rare occurrence [5–7].

Plain abdominal X-ray may reveal a cystic, gas-filled structure as it did in our patient. Frequently these studies are normal. Ultrasonography may also be a useful imaging modality; however the diagnosis is best established with CT imaging or contrast enema. Contrast studies will demonstrate a filling defect or luminal communication with the bowel. Colonoscopic examination will reveal the duplication cyst only if there is communication with the colon.

The management strategy of asymptomatic duplication cysts is not clearly established. Some authors favor routine resection with the goal of removing the potential for complications such as perforation, bleeding, obstruction, and malignant change [8]. The treatment of symptomatic colonic duplications is en bloc resection of the cyst and adjacent viscera. Occasional small cystic duplications can be excised without colonic resection if there is no compromise of blood flow to the adjacent intestinal segment. Sigmoid colonic duplications have also been resected via a laparoscopic approach [9].
